# Pedunculated Nasopharyngeal Mass: A Case of Squamous Cell Carcinoma

**DOI:** 10.7759/cureus.61917

**Published:** 2024-06-07

**Authors:** Raed Almutairi, Ali I Almania, Abdullah N AlHwiriny, Labeb M Sailan

**Affiliations:** 1 Otorhinolaryngology, King Fahad Specialist Hospital, Buraidah, SAU; 2 Medicine and Surgery, Qassim University, Malida, SAU; 3 Medicine, Qassim University, Malida, SAU

**Keywords:** tumors, otolaryngology-head & neck surgeons, nasopharyngeal mass, nasopharynx, nasopharyngeal squamous cell carcinoma

## Abstract

Nasopharyngeal carcinoma (NPC) is one of the rarest malignancies and carries a high risk of morbidity and mortality. The presentation of the disease depends on the stage and the anatomical relation of the lesion. In this case report, we present a case of a young female patient, who was found to have a pedunculated nasopharyngeal mass upon examination. The patient presented with nasal obstruction, which improved after surgical removal of the lesion. A histopathological examination of the resected mass revealed an undifferentiated squamous cell carcinoma type, which usually arises as an exophytic raised mass and not a pedunculated mass as in this case.

## Introduction

Nasopharyngeal carcinoma (NPC) is an epithelial malignancy from the nasopharyngeal mucosal lining. The tumor often arises in the nasopharynx from the pharyngeal recess (fossa of Rosenmüller) [1]. Compared with other cancers, NPC is relatively uncommon. According to the International Agency for Research on Cancer, in 2022, the incidence of NPC reached 1.3 per 100,000 people [2]. At the national level in Saudi Arabia, the national average age-standardized rate of nasopharyngeal cancer per 100,000 people over a study period of 10 years was 1.06, with the correlation between the age and incidence of the disease being noticed, with increased incidence with age [3]. The clinical presentation of patients with NPC depends primarily on the invasion of the primary disease and nodal involvement. Routes of invasion of the tumor include anterior extension into the nasal cavity, adjacent sinuses as maxillary sinus, or pterygoid fossa. Some lateral structures that could be affected by the invasion beyond the pharyngobasilar fascia are the parapharyngeal and infratemporal spaces and other structures of the skull, including the base, clivus, and intracranial structures if the invasion continues posteriorly and superiorly. There are many routes for possible invasion; hence, the clinical presentation will mostly correlate with the anatomical structures affected, and patient complaints vary accordingly, ranging from nonspecific (and possibly non-concerning) initially as nasal bleed or nasal obstruction, which is usually unilateral, and auditory symptoms, and possible cranial nerve palsies [4].

Regarding the macroscopic features, these tumors are usually observed with exophytic growth patterns in most cases, with less than 10% presenting as ulcerated lesions [5,6]. The natural history and treatment outcome for patients with these tumors have been correlated with clinical and, more recently, laboratory parameters. The prognosis has been clearly associated with the stage at presentation, including the size and extent of the primary tumor, degree of lymph node involvement, and the presence of distant metastases [7]. A Chinese study included 629 cases of NPC, wherein distant metastasis was found in 125; 95% of the distant metastases occurred within three years after completion of radiotherapy (i.e., 52% in the first year, 23% in the second year, and 20% in the third year), with bone metastasis being the most common site observed [8].

## Case presentation

**Figure 1:**
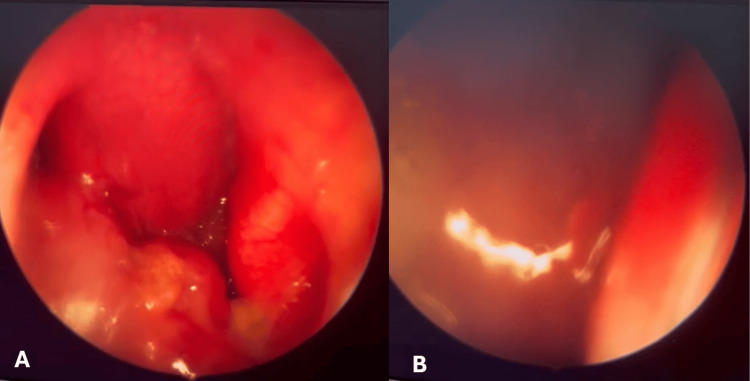
Nasal endoscope (A) A nasal endoscopic picture through the right side showing a pedunculated mass falling into the oropharynx with inspiration. (B) The mass moving up and obstructing the endoscopic view from the right choana with expiration.

We present a case of a 23-year-old female with no surgical or medical history, presenting to the otorhinolaryngology clinic on her first visit, complaining of progressive nasal obstruction that started eight months ago, along with snoring and choking while sleeping. Nasal endoscopy was performed in the clinic as a part of the examination of the nasal cavity and nasopharynx that revealed a pedunculated nasopharyngeal mass originating from the right side of the nasopharynx (Figure 1). The mass was moving up and down with breathing. Clinical examination of regional lymph nodes was negative for lymphadenopathy, although subsequent imaging with enhanced CT showed evidence of bilateral cervical lymph node involvement.

**Figure 2:**
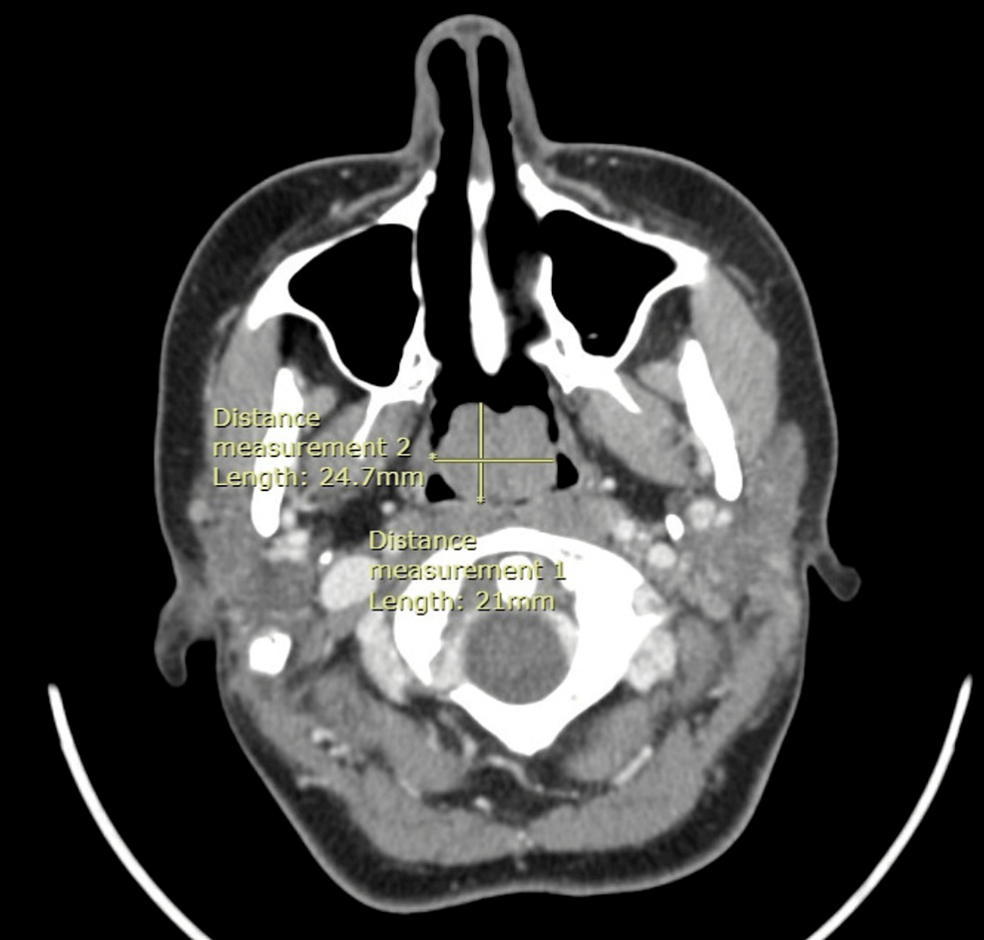
Axial CT scan The axial CT scan shows the nasopharyngeal mass with measurements of the size (approximately 2.47*2.1 cm).

Contrast CT was conducted, which showed a central nasopharyngeal relatively large polyp indenting the soft palate and significantly attenuating the nasopharyngeal airway, without locally aggressive imaging features. Bilateral cervical mildly prominent reactive lymph nodes were seen, at level II, reaching 12 mm (Figure 2).

**Figure 3:**
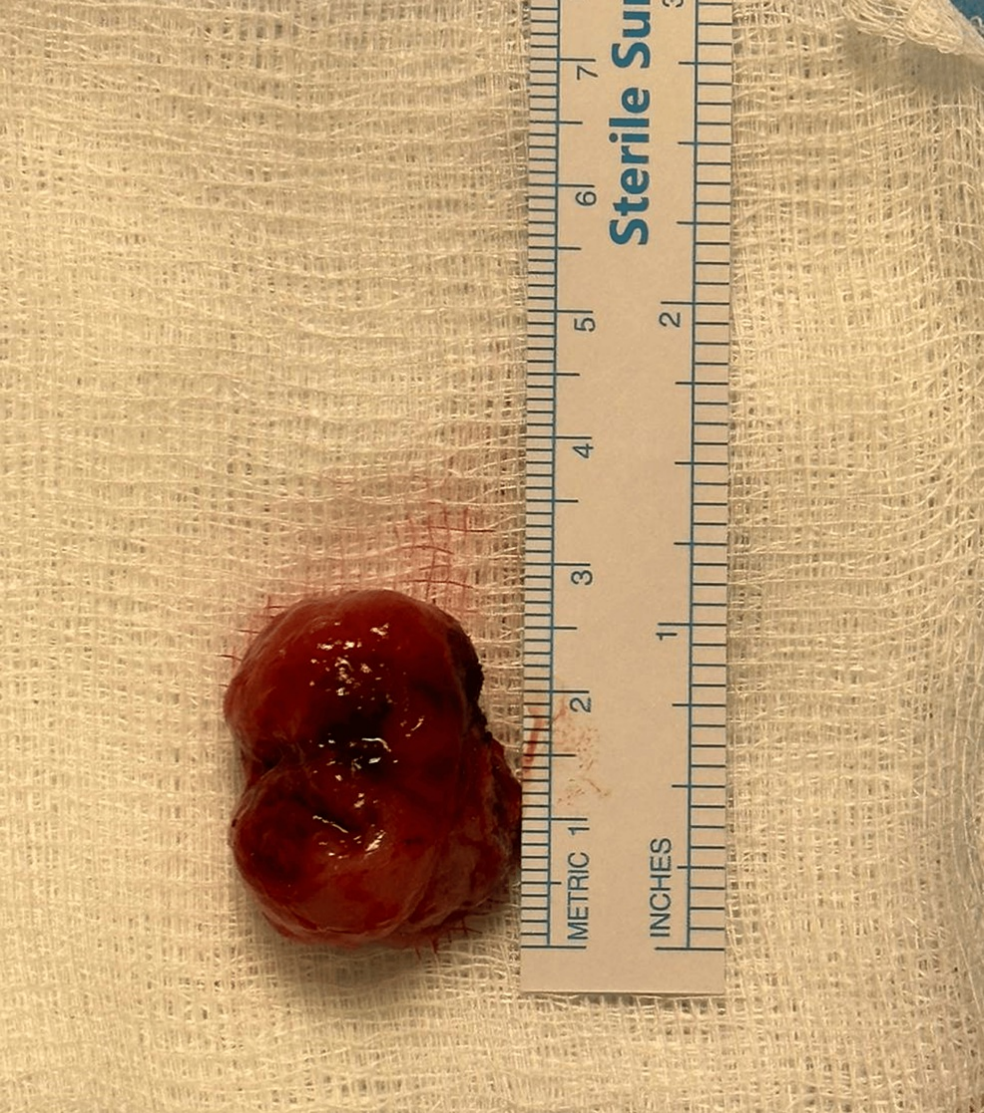
The excised nasopharyngeal mass

**Figure 4:**
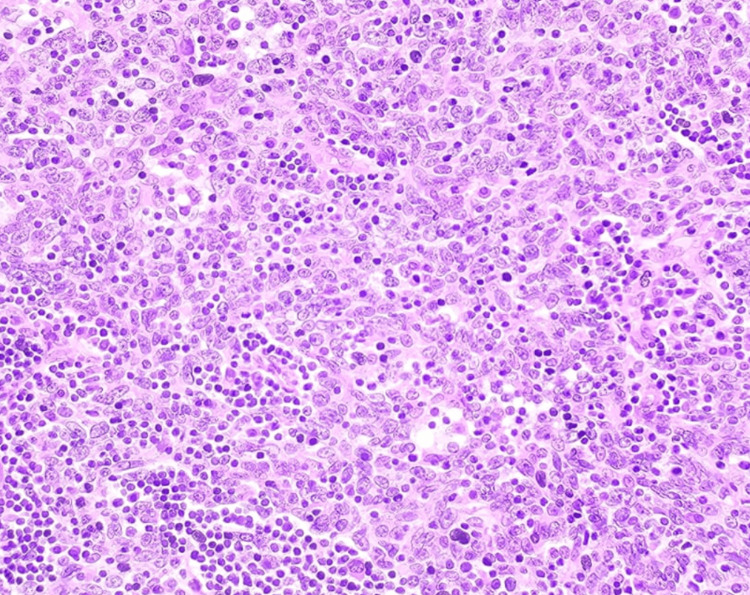
Histopathology slide with hematoxylin and eosin stain The fragment of tissue shows an invasive tumor composed of sheets, nests, and scattered large cells showing scant cytoplasm and enlarged nuclei with prominent nucleoli admixed with lymphoid cells.

**Figure 5:**
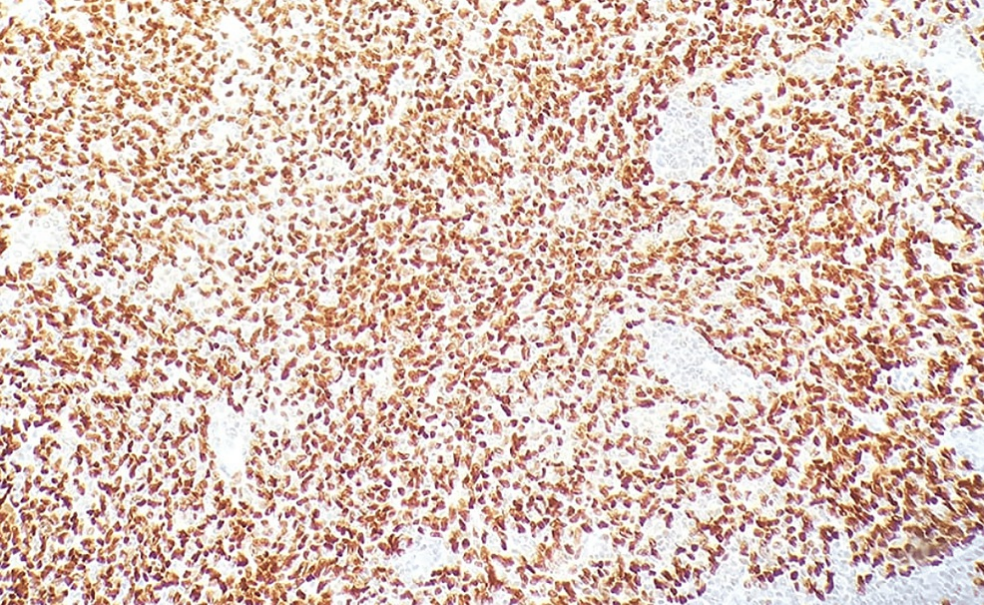
Histopathology slide with immunohistochemistry stain Positive p63 immunostaining.

Surgical excision of the nasopharyngeal mass was conducted under general anesthesia, via trans-oral technique, with the use of bipolar diathermy. The mass measured approximately 2.5 cm in its longest dimension (Figure 3), and the specimen was sent for histopathological diagnosis. Histopathology assessment revealed non-keratinizing squamous cell carcinoma, undifferentiated subtype (Figures 4-5). The patient was urgently referred to medical oncology. Subsequent assessment for distant metastasis was done with a chest-abdomen-pelvis CT with contrast and bone scan, and both investigations, fortunately, showed no evidence of distant metastasis.

**Figure 6:**
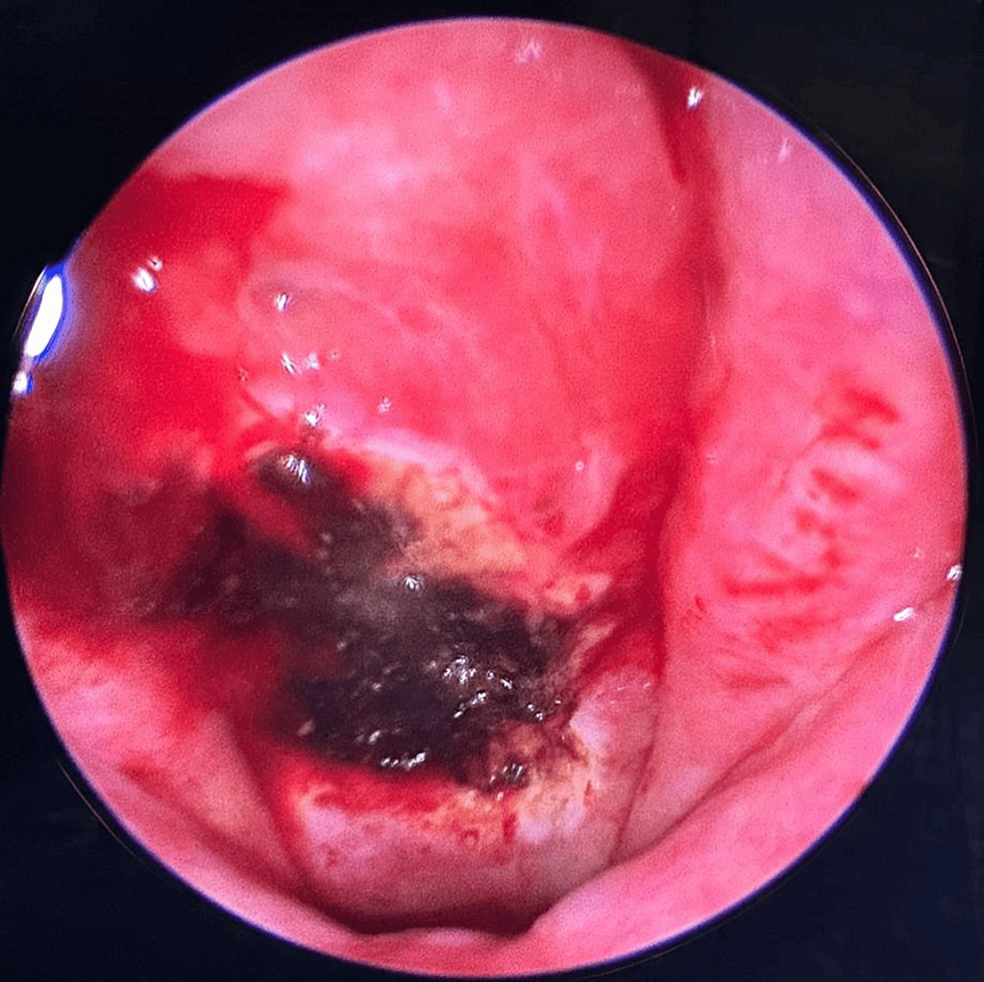
Excision site in the nasopharynx

Upon follow-up, two weeks after the mass excision, the patient reported an improvement in nasal obstruction symptoms. Nasal endoscopy of the nasopharynx excision site is observed in Figure 6.

## Discussion

Nasopharyngeal cancer is one of the rare cancers when compared to other types [2]. Nevertheless, there are certain geographical variations, with China having one of the highest rates of incidence of nasopharyngeal cancer. In Saudi Arabia, the incidence rate is approximately the same as the international rate but interestingly shows significant variation between different regions, with the Qassim region having the highest age-standardized rate [3]. Patients with NPC commonly experienced a delay in diagnosis, and Al-Rajhi et al. studied this issue. Their data showed that of the 307 patients included in the study, 88.6% were found to have delayed diagnosis, as their symptoms were present for at least three months before establishing a diagnosis [9]. The patient in our case had her first visit to the otorhinolaryngology clinic after eight months of symptoms onset. The presented case of nasopharyngeal mass is considered rare in this age of presentation (i.e., the early twenties), but the more interesting aspect of the case is the shape by which the mass has risen. As we mentioned before, the most common gross morphology of squamous cell carcinoma of the nasopharynx is exophytic, a discrete and raised nodule under the surface of the nasopharynx [5,6]. In our case, the mass was rather seen in a pedunculated shape as can be appreciated on both the neck CT scan and gross morphology of the mass; such presentation is rarely encountered in a patient with nasopharyngeal squamous cell carcinoma. The nature of the pedunculated mass being affected more by the pharyngeal movement with either respiration or swallowing could give rise to symptoms that vary in presentation with these activities, as with our patient. The differences in prognosis of pedunculated NPC need to be further studied. We did not find in the literature cases for nasopharyngeal squamous cell carcinoma where the masses presented as pedunculated, and this case could be the first case to be documented. Other histopathological types of nasopharyngeal mass can present such as this, for example, nasopharyngeal papillary adenocarcinoma and ex pleomorphic adenoma [10-12].

## Conclusions

It is important to know the common presentation of nasopharyngeal carcinoma, and the different possible presenting morphologies of the primary tumor upon examination of the nasopharynx. The physician's differential diagnosis should not be narrowed only based on the shape or morphology. Rather, a histopathological diagnosis should be the key to the final diagnosis and possible reassurance.
